# Editorial: Mitochondrial dysfunction affects mechano-energetic coupling in heart failure

**DOI:** 10.3389/fmmed.2024.1433102

**Published:** 2024-07-03

**Authors:** Jan Dudek, Julia Ritterhoff

**Affiliations:** ^1^ German Center for Heart Failure, Faculty of Medicine, University of Würzburg, Würzburg, Germany; ^2^ Molecular and Translational Cardiology, Department of Internal Medicine III, Heidelberg University Hospital, Heidelberg, Germany; ^3^ German Center for Cardiovascular Research (DZHK), Partner Site Heidelberg, Heidelberg, Germany; ^4^ Mitochondria and Metabolism Center, Department of Anesthesiology and Pain Medicine, School of Medicine, University of Washington, Seattle, WA, United States

**Keywords:** mitochondria, heart failure, metabolism, cardiomyopathy, mechano-energetic coupling

Heart failure (HF) is a continuously growing public health problem, affecting 1%–3% of the population (van Riet et al., 2016). Common causes of HF are hypertension, diabetes and obesity. HF is associated with metabolic dysfunction involving alterations in the choice of the preferred substrate, changes in the intermediary metabolism and defective energy and redox homeostasis. Increasing data indicate a direct link between cardiac metabolism and heart function. Mechanistically, metabolic changes affect cardiac function by causing epigenetic alterations, dysregulated signaling pathways and altered post-translational regulation (Bertero and Maack, 2018b; Lopaschuk et al., 2021; Ritterhoff and Tian, 2023). Conversely, increased pathological cardiac workload in HF can exceed energy supply-and-demand-matching and induce severe metabolic alterations (Bertero and Maack, 2018a). The goal of this Research Topic is to shed light on how cardiac metabolism and mitochondrial dysfunction affects the mechano-energetic coupling and ultimately, cardiac function.

Due to their central role in metabolism, mitochondrial are essential for cardiac function. Central mitochondrial functions including energy provision and redox homeostasis are regulated by Ca^2+^ signaling. In their review, Popoiu et al. summarize the detailed molecular mechanism, how mitochondrial function and myofilament contraction are linked. In the mitochondria, oxidative phosphorylation by the respiratory chain oxidizes NADH to NAD^+^ to convert ADP to ATP. ADP resulting from increases in workload is the substrate for the energy conversion at the respiratory chain and thereby accelerates mitochondrial respiration. Increased workload is controlled by elevated cytosolic Ca^2+^ levels, which are also transmitted into mitochondria via the mitochondrial calcium uniporter (MCU). Importantly, Ca^2+^ transmission into mitochondria stimulates Krebs cycle dehydrogenases in order to replenish NADH (Bertero and Maack, 2018a). This coupling of energy consumption and mitochondrial metabolism by Ca^2+^ not only regulates energy provision, but also maintains redox homeostasis under these conditions. Defects in redox homeostasis may provoke oxidative stress, which triggers maladaptive cardiac remodeling and HF progression (Nickel et al., 2015). Studies on an inherited cardiomyopathy called Barth Syndrome (BTHS) helped to reveal this concept. In BTHS, defects in the biogenesis of the mitochondrial phospholipid cardiolipin affect the mitochondrial membrane and integrated protein complexes, such as the respiratory chain, endangering energy metabolism. Other affected membrane protein complexes include MCU, which results in a lack of Ca^2+^ transmission into mitochondria. Decreased mitochondrial Ca^2+^ results in a deficient NADH replenishment and a redox mismatch. Redox induced arrhythmias and a lack of inotropic reserve in BTHS have been ascribed to MCU deficiency in BTHS (Bertero et al., 2021).

BTHS provides an interesting case, where structural alterations of the membrane and embedded protein complexes directly affect cardiac energy metabolism and redox homeostasis. To comprehensively study the membrane alterations in BTHS, Hachmann et al. analyzed lipid species in a mouse model of BTHS at 10 and 50 weeks of age, using electrospray ionization tandem mass spectrometry. Interestingly, alterations of lipid species were not constrained to mitochondrial lipids, but also involved phosphatidylcholine (PC), phosphatidylethanolamine (PE) and plasmalogen species. As membrane lipids are involved in multiple cellular processes, including signaling, this study lays groundwork for further studies, on how mitochondrial and cardiac function interconnect.

The development of increasingly complex cellular models of mitochondrial cardiomyopathies enables researchers to study the link of metabolism and cardiac function in detail. In their review, Rebs and Streckfuss-Bomeke discuss the use of stem cell-derived cardiomyocytes as disease models. A great challenge in this field is that iPSC-CM resemble an immature, neonatal status of cardiac cells in terms of their cellular and functional parameters, when compared to primary cardiomyocytes. Interestingly, recent advances in the maturation protocols use metabolic interventions to drive the maturation process.

The central role of nicotinamide adenine dinucleotide (NAD) in cardiac metabolism and redox regulation attracts increasing attention (Walker and Tian, 2018). In its reduced form NADH shuttles electrons from the oxidative metabolism for energy conversion at the respiratory chain. At the same time its oxidized form NAD^+^ is required as electron acceptor for dehydrogenases in the metabolism. Moreover NAD^+^ is a substrate for key metabolic signaling pathways, such as Sirtuin (SIRT) deacetylases and the poly (ADPribose) polymerase PARP1 (Luo and Kraus, 2012). Importantly, total myocardial NAD(P)H levels and the redox state are perturbed in HF and that NAD^+^ supplementation improves cardiac function in preclinical models of HFrEF and HFpEF (Lee et al., 2016). Dierickx et al. support this idea in a paper on cardiomyocyte specific knock-out of the REV-ERB nuclear receptors, which are components of the circadian clock. Loss of REV-ERBs in the mouse heart causes dilated cardiomyopathy and premature lethality. In this study, the authors report on supplementation with the NAD^+^ precursor NR to improve heart function to extend the lifespan of mice lacking cardiac REV-ERBs.

The availability of oxygen is a dominant regulator of energy conversion at the respiratory chain. The large size of cardiomyocytes, the densely packed cytosol and the large amount of oxygen consuming mitochondria affects even oxygen distribution, particularly in the center of the cell. In their review, Szibor et al. elucidate possible consequences of uneven oxygen distribution, which would result in local differences in energy conversion. Differently energized regions and ATP gradients in within the cell may create intracellular zones with different contractility, which may cause cardiomyocyte dysfunction affecting cardiac function. The authors discuss oxygen sensing mechanisms adapting respiratory chain activity and the direction and activity of the F_1_F_0_ ATPase. The authors propose that these regulatory mechanisms are necessary to adapt mitochondrial activity to local oxygen deprivation and prevent contractile dysfunction.

In conclusion, this Research Topic highlight the tight link of cardiac metabolism and function and how this regulatory network is rewired in cardiac disease. Better understanding of the molecular mechanisms opens the perspective of precise and personalized metabolic interventions in heart failure.
